# From the Cecum to the Sigmoid: Twisted Colon in the Pediatric Population

**DOI:** 10.7759/cureus.17974

**Published:** 2021-09-14

**Authors:** Raphael H Parrado, Nathan S Rubalcava, Katherine P Davenport

**Affiliations:** 1 Division of Pediatric Surgery, Medical University of South Carolina, Charleston, USA; 2 Division of Pediatric Surgery, University of Michigan, Ann Arbor, USA; 3 Division of Pediatric Surgery, Phoenix Children's Hospital, Phoenix, USA

**Keywords:** cecal volvulus, sigmoid volvulus, abdominal pain, intestinal obstruction, colon

## Abstract

Colonic volvulus (CV) is the third leading cause of colonic obstruction in adults. In infants and children, this is exceedingly rare, with only sporadic cases reported so far. We present two cases of CV to highlight the differences in etiology, presentation, diagnosis, and treatment of this condition.

The first patient is a 12-year-old boy with no previous surgeries who presented with four days of abdominal pain. Imaging showed a sigmoid volvulus that was decompressed endoscopically, and he was discharged. He had a contrast enema showing an abnormal rectosigmoid ratio. At the time of the rectal biopsy four weeks later, he was found to have a recurrence, at which point definitive operative treatment was pursued. The second patient is a 17-year-old boy who presented with five days of abdominal pain and CT findings concerning for ischemic volvulus. This prompted emergent operative intervention, where a cecal volvulus was discovered as the result of a congenital band. The band was divided without complication.

Pediatric CV is a rare condition that might be severe in some cases. High suspicion, prompt diagnosis, and treatment are essential to prevent early and long-term morbidity.

## Introduction

Colonic volvulus (CV) is a major cause of colonic obstruction in adults (3.4% of bowel obstruction cases in the United States); however, in the pediatric population, CV is exceedingly rare. The literature is limited to case reports and small case series, with the common point being a surgical emergency that can be life-threatening [1]. CV is defined as an acute torsion or kinking of any region of the colon on its own axis that can impair the mesentery and, conversely, the blood supply. It can progress to ischemia, necrosis, perforation, and sepsis, if not managed early [2]. We present two cases of CV presenting in different regions of the colon that conversely have a different presentation, diagnosis, and treatment.

## Case presentation

Case 1

**Figure 1:**
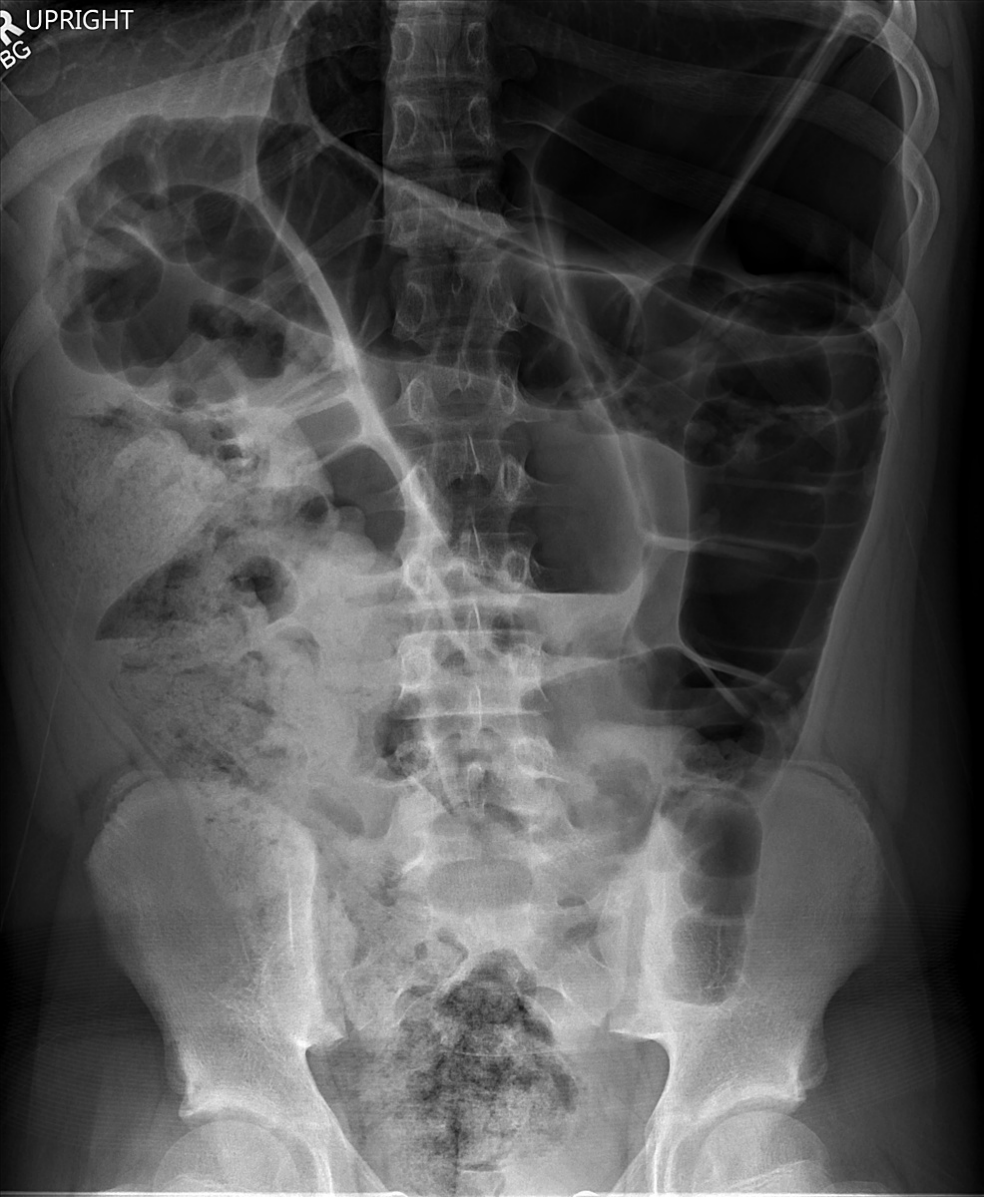
Abdominal X-ray at admission showing dilation, suggesting sigmoid volvulus. There is also moderate fecalization of the distal colon.

A 12-year-old boy presented with four days of abdominal pain associated with nausea and emesis. He had a history of chronic constipation (several years) and was not on any medications. On physical examination, his abdomen was soft and non-tender, and his vital signs were normal. The laboratory results showed a white blood cell count of 9.7 × 10^9^/L (with no left shift), and electrolytes were within normal limits. An abdominal radiograph showed dilation of the sigmoid colon with air-fluid levels concerning for sigmoid volvulus (Figure 1). A rectal tube was placed, and a decompressive colonoscopy was successfully performed without complications. He was discharged on hospital day two without any issues.

**Figure 2:**
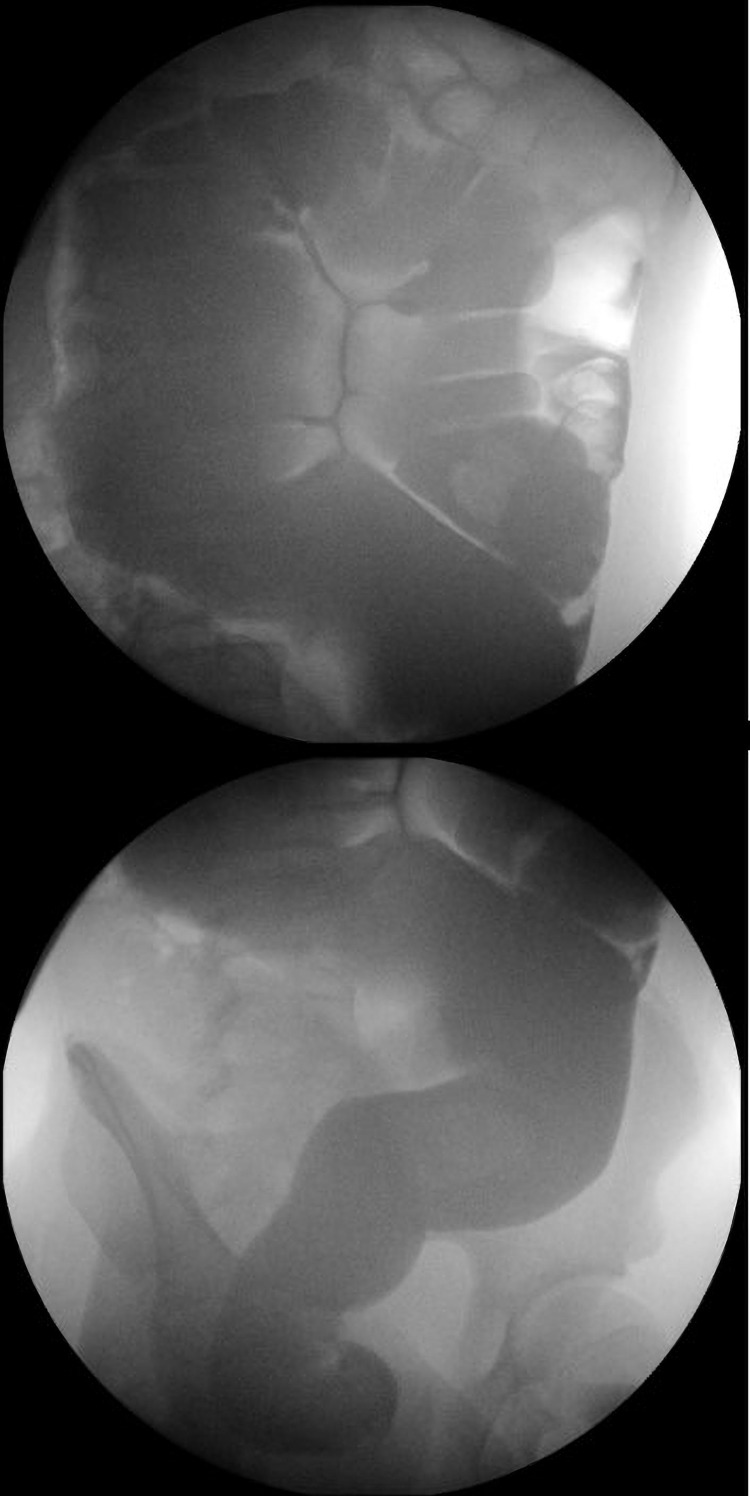
Contrast enema showing a redundant sigmoid colon with abnormal rectosigmoid ratio.

At the four-week follow-up, a contrast enema showed an abnormal rectosigmoid ratio (Figure 2). Due to the concern of Hirschsprung’s disease (HD), he was scheduled for an examination under anesthesia (EUA) and rectal biopsy. The morning of his procedure, he complained of abdominal bloating, which prompted an intraoperative X-ray and showed an incidental recurrence of the sigmoid volvulus. This was reduced using a rectal tube, and the following day, he underwent laparoscopic sigmoid colectomy (48.5 cm resected) with an intracorporeal end-to-end stapled anastomosis. There were no complications. The pathology report showed benign colonic tissue with ganglion cells; he has not had any issues on follow-up.

Case 2

A 17-year-old boy presented with five days of abdominal pain associated with dry heaving without emesis. He had a history of Kawasaki disease requiring multiple heart catheterizations for coronary artery aneurysms and a laparoscopic pancreatic cyst excision at age six. His vital signs were normal, and his abdomen was soft but tender in the right lower quadrant. White blood cell count was 9.9 × 10^9^/L (with no left shift), and the remainder of the blood and metabolic panel was normal.

**Figure 3:**
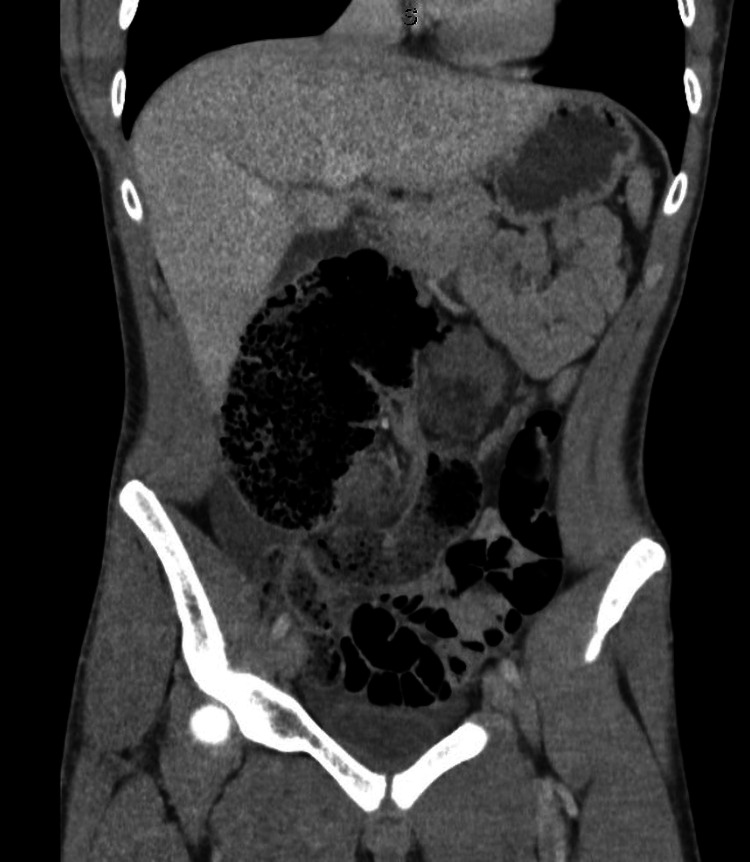
Abdominal CT showing a distended and medially located cecum with fecalization of the distal ileum and decompression of the ascending colon, all concerning for cecal volvulus.

**Figure 4:**
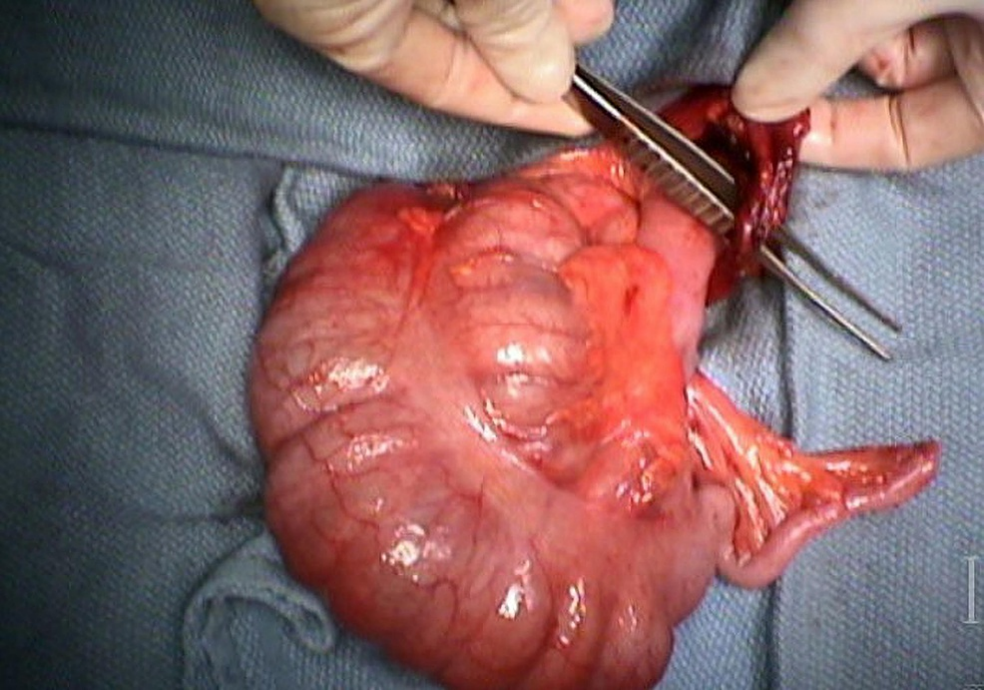
Operative image of the already reduced cecum. Forceps indicating the region where the congenial band and the axis of the torsion were encountered.

An abdominal CT with intravenous contrast showed findings concerning for cecal volvulus (Figure 3). He was taken immediately to the operating room for a diagnostic laparoscopy, where he was found to have torsion of the cecum caused by a congenital band (Figure 4). After the reduction and division of the band through a small laparotomy, an ileocecectomy with an end-to-end hand-sewn anastomosis was created. The patient recovered well and was discharged on postoperative day six. He reported no issues on follow-up.

## Discussion

CV is an extremely rare pediatric condition, limited to case reports, that results from an axial twist of the colon. In older adults, CV occurs most commonly in the sigmoid colon, while cecal involvement occurs more frequently in younger adults. However, in the pediatric population, CV can occur on either side. Most pediatric studies cite the cecum (75%) as the most common location for CV. A small case series evaluated patients with transverse CV and predisposing factors, including neurodevelopmental delay, as well as chronic constipation and dysmotility disorders [3]. A more notable association was observed with HD, as sigmoid volvulus has been reported in up to 0.6% of children with HD. More importantly, about 18% of children with sigmoid volvulus also have HD [4,5].

Pathologically, there are several hypotheses. One of them states that CV results from congenital bands that may predispose the colon to axial torsion, as shown in one of our cases. Another theory suggests that chronic stool accumulation due to constipation may lead to the stretching of the colonic ligaments and mesocolon, causing an increased risk of torsion. Furthermore, reported cases of sigmoid volvulus have shown redundant colon with elongated mesentery and a narrow base [6]. There have also been reports of a lack of fixation of some regions of the colon with a narrow mesenteric root. However, rather than being a form of malrotation, these tend to maintain the normal anatomic configuration that is often distorted in malrotation [3].

The presentation can be varied but often ranges from indolent to the more frequent presentation of abdominal pain, nausea, and bilious emesis. However, in a child with neurodevelopmental delay, CV might be presented with symptoms of constipation, making a delayed diagnosis of volvulus more likely. An abdominal radiograph might show a markedly dilated colon with absent distal gas, raising concern for obstruction. Given the low specificity of a plain abdominal X-ray, a contrast enema can usually show the narrowed, twisted colon with a “bird-beak’’ deformity. In the early phases, barium enema may help with decompression. A report of 42 cases showed that just half of the children had a barium enema prior to surgical treatment [6]. CT is another imaging modality that can better define CV. CT is another tool of diagnosis that will show dilated bowel with air-fluid level separated with septa and mesenteric swirling in some cases. It is important to note that in the setting of peritonitis or an unstable patient, minimal to no diagnostic imaging should be obtained, and operative treatment should be pursued.

As in adults, the literature describes colonoscopy as a tool for diagnosis, immediate relief, and preparation for surgical intervention. A report of 14 children with CV reported a recurrence rate of 57% after decompressive colonoscopy [7]. As mentioned previously, barium contrast enema can also be useful in the decompression of CV. It has a success rate of about 68%-79%, with an early recurrence of 10%-35% [7,8]. If unable to be reduced by either barium contrast or colonoscopy, operative intervention must be sought. 

Definitive treatment includes operative resection of the compromised bowel with primary anastomosis or fecal diversion through a colostomy with a latter takedown, depending on the degree of injury and peritonitis [9]. There have been reports of colopexy or sigmoidopexy; however, whether these additional measures are needed is still unclear [9,10]. Prognosis is related to the presence or absence of necrotic bowel. Mortality has been reported to be around 14%, mostly related to complications from shock and ischemia.

## Conclusions

CV is a rare condition in the pediatric population. It seems to be related to chronic constipation and HD. It can occur in any region of the colon. Plain abdominal X-rays are fine as an initial diagnostic tool; however, barium contrast enema and CT are the best modalities for confirming the diagnosis. Treatment is varied, and efforts should be made to develop standard practices for this condition. High suspicion, prompt diagnosis, and treatment are essential to prevent early and long-term morbidity.
